# A comparison of behavioural models explaining cervical cancer screening uptake

**DOI:** 10.1186/s12905-022-01801-2

**Published:** 2022-06-16

**Authors:** Jyoshma Preema Dsouza, Stephan Van den Broucke, Sanjay Pattanshetty, William Dhoore

**Affiliations:** 1 School of Public Health, Psychological Research Institute, Université Catholique de Louvain, 1348 Ottignies Louvain-la-Neuve, Belgium; 2 Prasanna School of Public Health, Manipal Academy of Higher Education, Manipal, Udupi, Karnataka 576104 India

**Keywords:** Cervical cancer, Low-middle-income countries, Screening behaviour, Health behaviour, Psycho-social factors, India, Women

## Abstract

**Background:**

Cervical cancer represents a very high burden of disease, especially in Low- and Middle-income economies. Screening is a recommended prevention method in resource-poor settings. Cervical cancer screening (CCS) uptake is influenced by various psycho-social factors, most of which are included in behavioural models. Unlike demographic characteristics, these factors are modifiable. While few studies have compared these models in terms of their capacity to predict health behaviour, this study considers three health behaviour theories to assess and compare the predictors of CCS behaviour and intention.

**Methods:**

A survey was conducted among 607 sexually active women in the South Indian state of Karnataka. Data was collected regarding socio-demographic factors, health literacy, knowledge on CCS, and the socio-cognitive factors related to CCS that are represented in the Health Belief Model (HBM), Theory of Planned Behaviour (TPB) and Theory of Care-Seeking Behaviour (TCSB). Logistic regression analyses tested to what extent each of the theoretical models explained cervical cancer screening (CCS) intention and regular screening behaviour, comparing the variance explained by each of the models.

**Results:**

CCS intention was best explained by the TPB, followed by the HBM. Of the constructs included in these models, positive attitude towards the screening procedure and perceived benefits contributed most significantly to screening intention, followed by fear, anxiety or embarrassment related to the disease or screening procedure, and context specific barriers.

**Conclusion:**

Health behavioural models such as the TPB and HBM can help to identify the main socio-cognitive factors explaining the intention of women to participate in CCS. As such, they can inform interventions to target specific determinants of screening intention and behaviour, and enhance their effectiveness by addressing women’s screening attitude, perceived benefits, and emotions as well as reducing context specific barriers to screening.

## Background

Cervical cancer is one of the most common cancers among women, after breast, colorectal and lung cancer. In 2020, nearly 0.6 million women developed cervical cancer, and 0.3 million died of the disease [1]. Cervical cancer poses causes a significant impact on the economic [2], psycho-social status [3] and overall quality of life [4] of individuals and also affects a country’s economy. More than three-fourths of the overall deaths due to cervical cancer happen in Low- and Middle Income countries (LMICs) [5, 6]. In these countries, cervical cancer is one of the leading cause of premature death or life years spent with disability [7]. This is also the case for India, which in 2018 had the highest age-standardized incidence of cervical cancer in Western Asia, accounting for up to a quarter of the total cervical cancer deaths [8, 9] In India, Cervical cancer represents 30% of all cancers among women [9], contributes to about 7.8% of the total cancer Disability Adjusted Life Years [10], and poses a huge burden on the country’s economy.

Unlike some other cancers, cervical cancer can be prevented, with primary prevention through vaccination and secondary prevention through screening representing the two main strategies. Early detection of the Human Papilloma Virus (HPV) that causes the infection in the cervix can help treat the infection, thus preventing its progression to cancer. Screening and early treatment to prevent the disease can thus lead to a better prognosis and reduce the morbidity and mortality due to the illness [11]. Several cost-effective strategies combining human papillomavirus vaccination, cervical screening, and treatment have proven to be suitable for low-resource settings [12] and found effective in LMICs like India [13]. Methods like HPV testing could replace expensive colposcopy procedures in LMICs where resources are scare and the burden of cervical cancer is high [14]. In addition to its excellent sensitivity and long-lasting negative predictive value, HPV-based screening can successfully be implemented as a “see-and-treat” approach where screening, triage, and treatment are provided at the same visit, or be performed on self-collected specimens. This provides substantial benefits for its implementation in remote areas where women must travel long distances for screening and treatment, and where health care provider resources are limited. However, various factors affect the implementation of such strategies.

In India, the high cost of the vaccine, its inability to protect against some strains of the virus, uncertainties related to its protection period and doubts regarding its acceptability among people make it difficult to implement vaccination on a national scale, whereas cost-effective screening methods have been piloted and proven feasible and effective [15–17]. However, the participation of women in screening programs is generally low. To have an impact on the prevalence of cancer, more women need to be screened. Therefore, it is important to identify the factors that can enhance women’s participation in cervical cancer screening, especially in LMICs where the burden of disease is high.

In addition to various well-documented demographic, socio-economic and cultural factors that influence the screening uptake by women [18], the importance of psychosocial factors such as beliefs, perceptions and emotions are to be noted. These psychosocial factors are generally easier to modify than socio-demographic or cultural determinants like education, socio-economic status or cultural habits, which makes them interesting as potential targets for interventions. To help identify the psychological factors that impact on screening uptake, use can be made of health behaviour theories, which explain human health behaviour based on a combination of well-researched and validated psycho-social concepts and their interactions.

### Health behaviour theories

**Fig. 1:**
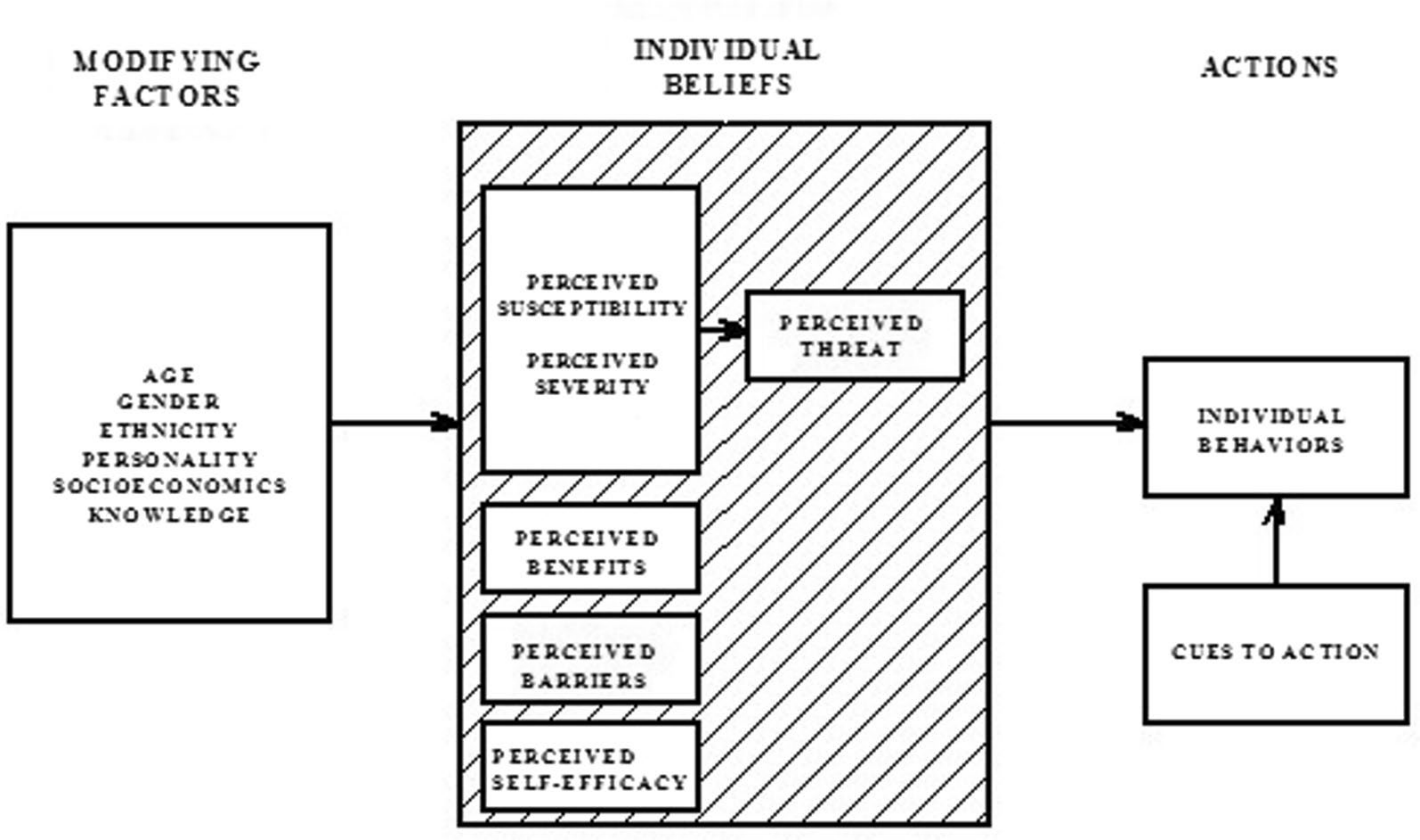
Health Belief Model components. *Source*: Champion and Skinner, 2008, pg.48

Several behavioural theories hold that health behaviours are to a significant extent determined by personal beliefs. One of the most well-known of these theories is the *Health Belief Model* (HBM), developed in the 1950s [19]. This model has been applied extensively to a range of behaviours related to health [20–22]. It postulates that individuals will execute preventive health behaviour if they believe that (1) They are at risk of developing a given disease or condition, (2) The consequences of the disease are severe or life-threatening, and (3) A specific behaviour can be performed to prevent the disease, of which the benefits outweigh any barriers (Fig. 1). Several studies have used HBM to explain CCS [23] and a few have predicted CCS uptake [24]. Moreover, the addition of certain dimension to the basic HBM model, such as perceived self-efficacy (i.e., the belief that one is capable of performing the behaviour), intentions, and cues to action, improves its capacity to explain preventive behaviour [25].

**Fig. 2:**
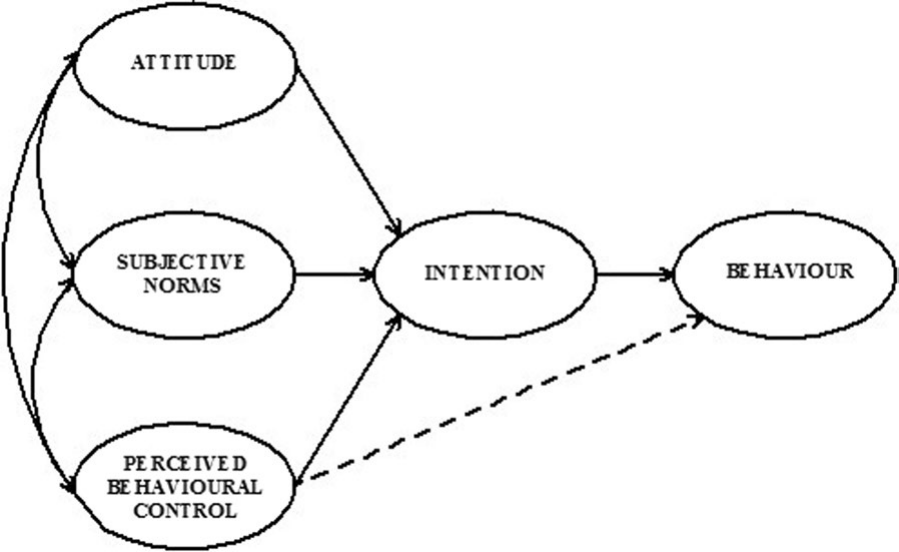
Theory of Planned Behaviour components. *Source*: Ajzen, 1991

Another often-used health behaviour model is the *Theory of Planned Behaviour* (TPB), developed by Ajzen [26] as an extension of the Theory of Reasoned Action [27]. This model implies that individuals are more likely to perform a certain behaviour if they have the intention to do so, and that this intention in turn is dependent on three types of beliefs: (1) That the behaviour will lead to positive outcomes (i.e., behavioural beliefs); (2) That it is supported by significant others (i.e., normative beliefs); and (3) That one is capable of performing it (i.e., self-efficacy beliefs) (Fig. 2). Over the years, a very extensive body of research, including several meta-analyses, has proven the validity of the TPB to predict behaviour, and several studies have shown its effectiveness to predict CCS intention [28, 29].

**Fig. 3:**
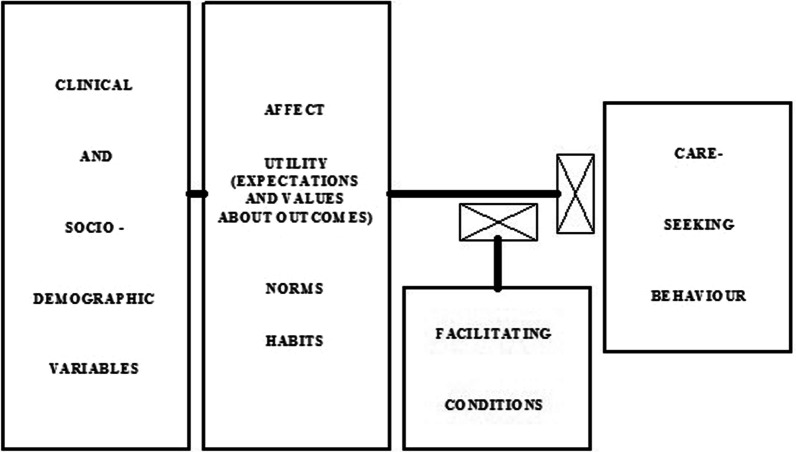
Theory of Care Seeking Behaviour components. *Source*: Lauver 1992

A third, less well-known health behaviour theory is the *Theory of Care-Seeking Behaviour* (TCSB). This model was developed by Lauver [30] based on Triandis’ Theory of Interpersonal Behaviour [31], and includes four main constructs (Fig. 3). It proposes that individuals are likely to carry out a preventive behaviour if they (1) generally follow healthy practices, (2) have no negative emotions of fear, anxiety or embarrassment about performing the behaviour but instead have a positive attitude about its outcome, and (3) feel supported by significant others to do so; and (4) when the socio-demographic situations are conducive. This theory has been used to explain people’s behaviour on recommended screening tests [32] and is thus also potentially suitable to understand cervical cancer screening behaviour.

Although all these three theories have been used to explain health related behaviours and predict intentions to perform health-enhancing behaviours, there is uncertainty as to which theory is better at explaining intentions or behaviours and which constructs within the models are better predictors [33]. Identifying the best suitable theories would thus provide a better basis to use them for exploring the determinants of health behaviour. Besides, there are very few studies that have applied these models to cervical screening uptake. To our knowledge, only one study compared the HBM and TPB with regard to predicting CCS behaviour [34], but thus far no studies compared these models with others in terms of predicting CCS uptake or intention.

The present study aimed to address this issue by (1) identifying the main psychosocial determinants of cervical cancer screening intention among women using three theoretical models (i.e., the HBM, TPB and TCSB; and (2) by comparing the potential of the different models and their respective components to explain women’s intentions to participate in CCS. Since each of these three models includes distinct theoretical constructs, a comparison of the models’ capacity to explain CCS, and of the relative contribution of each construct to explain screening uptake can help to identify the key factors that influence CCs intention. As such, the study involved five basic constructs derived from the HBM (perceived susceptibility, perceived severity, perceived benefits, perceived barriers and perceived self-efficacy); three constructs from the TPB (attitude, subjective norm, and perceived behavioural control); and four constructs derived from the TCSB (affect, utility, subjective norms, and habits).

## Methods

### Study design and setting

A cross-sectional survey was conducted as part of a larger survey among sexually active women dwelling in Karnataka, a southern state of India, to explore determinants of screening uptake. The state has a population of 61.09 million with nearly 50% of the females aged between 15 and 44 years. Nearly 62% of the population lives in rural areas. The literacy rate of females is about 68%. Cervical cancer contributes to 13% of the cancers [9] and the average cervical cancer examination rate is 0–10% among women [35]. A semi-structured questionnaire was designed based on the theoretical constructs of HBM, TPB and TCSB and administered to the participants in person by the community health workers after obtaining informed consent. All methods were carried out in accordance with relevant guidelines and regulations. The present paper only presents the results regarding the determinants of screening uptake.

### Data collection

Data were collected with the help of accredited social health activists (ASHAs) willing to assist in the survey. ASHAs are community workers selected through a rigorous process from the community, and trained regularly to work as an interface between the community and the public health system. They are females aged between 25 and 45 years and usually receive incentives for specific activities related to national health programs. To ensure the quality of the survey data, the ASHAs workers were trained in the data collection procedure and a set of written instructions was provided with a possibility to contact the researcher whenever required. The data collection took place under the supervision of the researcher.

### Sample size and sampling method

The sample size of 764 for 50% response rate was calculated using Cochran’s formula Sample size = *Z*^*2*^ (p*q)/d ^*2*^, where the estimated predicted variance was set at 50%, with a 5% margin of error and 1.96 as the critical value(Z) for 95% confidence level. The predicted variance of 50% was based on the population proportion (i.e., *p* = 0.5 yields an adequate sample to represent the population), since no similar studies existed for the given population.

The study aimed to include a representative sample concerning accessibility to screening centres. In the first step cervical cancer screening centres with affordable facilities were identified across the districts. In the second step, two regions (an accessible region that had access to the screening centre and an inaccessible region that did not have access to the screening centre) were identified. In the final step, participants were approached from the two regions using consecutive sampling approach and data was collected with the help of the community health workers who approached participants and provided the questionnaire to be completed. All women aged 20–60 years, accessible at the time of data collection, able to read or understand Kannada or English could be included in the study. Women who stated having had cervical cancer were excluded from the study. Ethical clearance was obtained from the KMC Institutional ethics committee.

### Participants

The final sample consisted of a total of 607 women. The participants’ mean age was 36 ± 8 years, with the largest group aged between 31 and 45 years (58.5%), followed by 20–30 years (28.3%), and 46–60 years (13.2%). Their mean income was $ 294 per month. About one-fourth of the respondents (24%) had not completed secondary education, and 54% of them were unemployed. The vast majority (97%) had not been trained in a healthcare profession, and only 37% were able to use the internet to find health-related information. A large majority of nearly 80% of the participants did not have health insurance, and of those who did not have insurance, nearly 42% claimed to have had difficulty spending for routine health care check-ups. Also, for a majority of the participants (80%) decisions regarding their healthcare expenditure was taken by their partners or family members.

### Questionnaire

The survey questionnaire was developed based on a systematic review [36], a qualitative study [18] and a validation study conducted in the context [37]. In addition to socio-demographic characteristics, it measured the key 5 constructs of the HBM (perceived susceptibility, perceived severity, perceived benefits, perceived barriers and perceived self-efficacy), 3 constructs of the TPB (attitude, subjective norm and perceived behavioural control), and 4 constructs of the TCSB (affect, utility, norms and habits). The tool also consisted of questions that explored knowledge about disease and screening, CCS behaviour and CCS intention.

The resulting questionnaire consisted of five sections. The first section explored participants’ demographic and background characteristics including age, income, education and employment status, training in health care profession, health insurance status, health care expenditure related decision-making ability etc. The second section consisted of 16 items and explored knowledge pertaining to cervical cancer (cause, risk factors, clinical features) and screening (screening test and knowledge about regular screening) which were measured on a continuous scale. Expected screening facilitators like having had symptoms, having heard of cervical cancer screening, knowing someone with the disease were also added. The KR-20 was 0.84 for 16 knowledge questions, which was well above the acceptable limit of 0.5 showing good reliability [38].

The third section measured the variables based on the health behaviour theories. It consisted of 38 items to be scored on 5-point Likert scales ranging from ‘strongly agree’ to ‘strongly disagree’. Twenty four items measured the HBM-based dimensions of perceived susceptibility (2 items, ‘I feel I can get cervical cancer’, ‘I feel I am at a higher risk to get cervical cancer’), perceived severity (2 items, ‘Cervical cancer is a very serious health problem’, ‘Cervical cancer cannot be easily cured’), perceived benefits (3 items, e.g. ‘Screening can help early diagnosis and treatment becomes easy’, ‘Having regular tests makes me worry less’), perceived barriers (18 items, e.g. I am shy), and self-efficacy (8 items, e.g. I am able to undergo screening even if my husband or family disagree with my decision). The Cronbach’s α for perceived susceptibility, perceived severity, perceived benefits, perceived barriers, and self-efficacy was 0.682 (Spearman-Brown = 0.65), 0.652 (Spearman Brown = 0.62), 0.703, 0.869 and 0.935, respectively. Eighteen items measured the TPB-based dimensions of attitude (5 items, e.g., ‘Screening takes too much time’, ‘Screening causes pain’), subjective norms (5 items measuring injunctive norms like the opinion of one’s partner, family members and significant groups, and descriptive norms such as screening by significant people), and perceived behavioural control (PBC) (8 items, e.g. ‘How sure are you that you can get your cervical screening test, even if the test might be painful?’). Cronbach Alphas for the attitude, subjective norms and PBC scales were 0.823, 0.849 and 0.935, respectively. Fourteen items measured the dimensions of the TCSB: affect, or fear, anxiety and embarrassment related to the screening procedure and outcome (5 items, e.g., ‘I am afraid to have a cervical examination test because I don’t know what will happen to me’), utility or benefits of screening (3 items, e.g., ‘Having cervical examination test is the best way for cervical cancer to be diagnosed early’), subjective norms (5 items, e.g., ‘I do not want to do the test because my husband won’t let me do so’) and habit (1 dichotomous variable (yes/no) asking for general routine screening behaviour). Cronbach α values for the affect, utility and subjective norms scales were 0.714, 0.703 and 0.849, respectively. TPB based items included 5–8 items to explore each of its construct: Attitude, Subjective norms, and Perceived behavioural control (PBC). Five items like ‘Screening takes too much time ‘Screening causes pain’ measured an individual’s attitude to the screening process. Five items explored subjective norms or social influence. It included injunctive social norms (opinion of partner, family members and significant groups) and descriptive norms (screening by significant people). Perceived behaviourαal control, equivalent to Self-efficacy in HBM is one’s confidence to cope-up with the barriers to uptake screening. PBC was measured using 8 items. The Cronbach’s α for Attitude, Subjective norms and PBC were 0.823, 0.849 and 0.935 respectively. Theory of care-seeking behaviour included 14 items to measure its constructs: Affect, Utility and subjective norm. Five items related to fear, anxiety and embarrassment related to the procedure and outcome explored affect. Three items measured Utility or benefits of screening and five items explored subjective norms. One item explored general routine screening behaviour and included exploring ‘habit’ as a dichotomous variable. The Cronbach’s α for affect, Utility and subjective norms were 0.714, 0.703 and 0.849, respectively.

The fourth section included a valid and reliable health literacy measuring tool consisting of 16 items [37] and the last section consisted of three items measuring the participants’ CCS behaviour, asking them to indicate past screening behaviour, regular participation and intention to participate in CCS. Each of these were to be scored on a dichotomous scale (yes/no).

### Data analysis

Data analysis was done in SPSS version 25.0. Frequencies with percentages and mean scores with standard deviations were provided as descriptive statistical measures. In the first step, Chi-square was used as bivariate analysis to find the association between categorical independent variables and CCS intention as dichotomous outcome variables. Independent t-tests were performed to measure the differences in the continuous variables between groups with or without the intention to and participation in screening. In the second phase, a series of logistic regressions were performed to test several models. In the first model, only the socio-demographic variables that had been found significant (*p* < 0.05) in the bivariate analysis were regressed onto the ‘intention to participate in CCS’. For the second, third and fourth model, the constructs of the HBM, TPB and TCSB, respectively, were added to the sociodemographic variables to assess the increase in variance in screening intention and behaviour explained by each model, with adjustments made for the socio-demographic variables.

## Results

### Knowledge and experience with cervical cancer

Of the 607 women who participated in the survey, approximately 59.5% reported having had symptoms related to cervical cancer in the past. Nevertheless, the overall knowledge about cervical cancer and screening was low. A majority had not heard of cervical cancer (62%) or had not known anyone with the disease (87%). The median knowledge score was 3.5 (on a maximum of 15). Nearly 90% of the participants were unaware that the virus (HPV) causing the infection and leading to cervical cancer is transmitted through sexual contact. While more than three fourth of the participants claimed that the screening centres were accessible (79%), a large majority (95%) were unaware of the screening test and the need for regular screening. Nearly 80% of the participants had limited health literacy (Table 1).

**Table 1:** Socio-demographic and facilitating conditions of CCS intention

Socio-demographic variables				
	Total (N = 607)	CCS		*p*
		No intention (n = 358)	Intention (n = 249)	
Age (Mean, SD)	36.3 (8.2)	36.9 (8.4)	35.4 (7.8)	*NS*
Income (in thousand INR) (Mean, SD)	22.08 (10.4)	20.42 (9.62)	24.45 (11.04)	< 0.001
Employment (%)				
Unemployed	329 (54)	209 (58)	120 (48)	0.010
Employed	278 (46)	149 (42)	129 (52)	
Education level (%)				
Secondary education not completed	145 (24)	103 (29)	42 (17)	0.001
Secondary education completed	462 (76)	255 (71)	207 (83)	
Training in HC profession (%)				
No	586 (97)	346 (97)	240 (96)	*NS*
Yes	21 (3)	12 (3)	9 (4)	
Health insurance (%)				
No	484 (80)	301 (84)	183 (73.5)	0.01
Yes	123 (20)	57 (16)	66 (26.5)	
Easiness of HC expenditure (%)				
Difficult	238 (39)	147 (41)	91 (36.5)	*NS*
Easy	369 (61)	211 (59)	158 (63.5)	
Routine Health check-ups (%)				
No	364 (60)	212 (59)	152 (61)	*NS*
Yes	243 (40)	146 (41)	97 (39)	
Healthcare expenditure decision- making (%)				*p*
Others	485 (80)	307 (86)	178 (71.5)	< 0.001
Woman herself	122 (20)	51 (14)	71 (28.5)	
Facilitating conditions				
Accessibility to screening centre (%)				
Inaccessible	126 (21)	98 (27)	28 (11)	< 0.001
Accessible	481 (79)	260 (73)	221 (89)	
Had symptoms				
No	361 (59.5)	206 (57.5)	155 (62.2)	*NS*
Yes	246 (40.5)	152 (42.5)	94 (37.8)	
Heard of CC				
No	372 (61)	227 (63)	145 (58)	*NS*
Yes	235 (39)	130 (37)	104 (42)	
Known someone with cervical cancer (%)				
No	524 (86.5)	314 (88)	211 (85)	*NS*
Yes	83 (13.5)	44 (12)	38 (15)	
Health literacy level (%)				
Limited	478 (79)	304 (85)	174 (70)	< 0.001
Adequate	129 (21)	54 (15)	75 (30)	*NS*
Knowledge about CC (Mean, SD)	3.9 (3.4)	3.6 (3.18)	4.1 (3.4)	*NS*
Knowledge about CC screening				
Poor	576 (94.9)	349 (97.5)	227 (91.2)	0.05
Good	31 (5.1)	9 (2.5)	22 (8.8)	

*p values from T-test, Chi-square test or Fisher’s exact test where applicable*

### Screening behaviour and intentions

Only 10% of the participants (n = 58) reported to have undergone screening in the past, and only 1% (n = 8) reported to have been undergoing screening regularly. About 41% (n = 249) had a positive intention to undergo screening in the future.

### Correlates of CCs intention

The results of the bivariate analysis of the association of socio-demographic variables, facilitator variables, knowledge, and health literacy with CCS intention are given in Table 1. The intention to participate in CCS is associated with having a health insurance (OR = 1.9) and being able to make health care related decisions for themselves (OR = 2.4; p < 0.05). Individuals with adequate health literacy also have significantly higher odds (OR = 2) of having a positive screening intention. Furthermore, there is a significant association between CCS intention and accessibility to screening centres (OR = 2), and knowledge about screening (OR = 3.7). In contrast, having heard of cervical cancer, having had symptoms or knowing someone with the disease are not associated with screening intention.

Correlations between the constructs of the health behaviour theories are given in Table 2, showing mild to moderate correlations within the constructs of HBM, TPB and TCSB. Within the HBM, self-efficacy was positively correlated with susceptibility and severity was correlated with perceived benefits. Within the TPB, attitude was positively correlated with subjective norms but negatively correlated with perceived behavioural control, indicating that individuals with higher perceived behavioural control had a less negative attitude towards screening and those with higher attitudinal barriers also perceived social norm as less positive. Among the TCSB constructs, individuals with higher affect scores (i.e., those with higher barriers related to fear, anxiety, embarrassment etc.) had more positive subjective norms and lower perceived benefits of the screening procedure. Additionally, we also found that, past cervical cancer screening behaviour was not related to screening intention.

**Table 2:** Mean scores on health behaviour constructs for women with and without screening intention, and correlations between constructs

Health belief model							
	No CCS intention mean (SD)	CCS intention mean (SD)	*t-value*	Perceived susceptibility	Perceived severity	Perceived benefit	Perceived barriers
Perceived susceptibility	5.26 (1.4)	5.30 (1.4)	− 0.30				
Perceived severity	6.73 (1.31)	6.84 (1.42)	− 0.10	0.05			
Perceived benefit	10.96 (1.86)	11.57 (1.38)	− 4.39**	− 0.05	0.34**		
Perceived barriers	51.61 (5.01)	49.39 (6.07)	4.92*	0.03	0.03	− 0.07	
Perceived self efficacy	4.84 (2.61)	4.85 (2.70)	− 0.06	.20**	0.02	0.12**	− 0.07

Theory of planned behaviour					
	No CCS intention mean (SD)	CCS intention mean (SD)	*t-value*	Attitude	Subjective norm
Attitude	23.41 (2.34)	22.25 (2.76)	5.55		
Subjective norm	8.07 (2.14)	7.42 (2.03)	3.76	0.50**	
Perceived behavioural control	19.14 (4.65)	19.42 (4.91)	− 0.70	− 0.16**	− 0.04

Theory of care seeking behaviour					
	NO CCS intention mean (SD)	CCS intention mean (SD)	*t value*	Affect	Utility
Affect	12.71 (1.65)	12.05 (1.86)	4.60		
Utility	10.96 (1.86)	11.57 (1.38)	− 4.39**	0.03	
Subjective norms	8.07 (2.14)	7.42 (2.03)	3.76	0.35**	− 0.21**

*t value from independent test*

*r* = *Pearson’s correlation coefficient*

**p value < 0.001, *p value < *0.05*

Independent t-tests of the differences between women with and without an intention to participate in screening on the constructs of the three theoretical models (Table 2) indicate that there is a significant difference in the scores of perceived benefits and perceived barriers of HBM, and of utility and habits of TCSB. In contrast, women who intend to participate in screening and those who don’t intend to do so do not differ significantly on perceived susceptibility, perceived severity, self-efficacy, affect, subjective norms, or any of the constructs of the TPB.

### Prediction of screening intention

Binary logistic regression analysis was conducted with each of the models to assess the predictors of CCs intention and CCS behaviour one at a time, to be able to compare the variance explained by each of the models in the outcome.

Binary logistic regression analyses (Table 3) show that a model containing the socio-demographic variables of ‘having health insurance’, ‘being able to make healthcare-related decisions by themselves’, ‘accessibility to screening centre’, ‘health literacy status’ and ‘knowledge about screening’ has a good fit (Hosmer–Lemeshow goodness-of-fit χ^2^ = 1.694, df = 5, p = 0.890) and explains 16% of the variance in screening intention (R^2^ = 0.158, adjusted R^2^ = 0.150). The addition of the HBM constructs of perceived susceptibility, perceived severity, perceived benefits, perceived barriers, and self-efficacy, over and above significant socio-demographic variables, also has a good fit (Hosmer–Lemeshow goodness-of-fit test: χ^2^ = 4.61, df = 8, p = 0.79) and improves the explained variance by 2.8 to 19% (R^2^ = 0.192, Adjusted R^2^ = 0.178), when adjusted for other factors. Of the variables of the model, perceived benefits (β = 0.191, *p* < 0.005) and perceived barriers (β = − 0.048, *p* < 0.05) are significant predictors of screening intention, with higher perceived benefits about screening and lower perceived barriers being associated with a higher intention. Perceived susceptibility, perceived severity and self-efficacy do not contribute significantly to the prediction of screening intention. Furthermore, adding past screening behaviour to the model does not increase the explained variance (R^2^ = 0.195, adjusted R^2^ = 0.177), so controlling for past behaviour in the model does not alter the effect of perceived benefits and perceived barriers.

**Table 3:** Log-regression of the HBM, TPB and TCSB predicting CCS intention

	OR	95 CI	*P* value	OR	95 CI	*P* value	OR	95 CI	*P* value	OR	95 CI	*P* value
Health insurance	1.85	1.21–2.84	0.004	1.79	1.16–2.77	0.008	1.85	1.20–2.86	0.005	1.98	1.26–3.09	0.003
Healthcare-expenditure decision making	2.27	1.48–3.47	< 0.001	2.05	1.31–3.20	0.002	2.02	1.28–3.17	0.002	1.82	1.15–2.87	0.009
Accessibility to screening centre	2.97	1.84–4.82	< 0.001	2.8	1.71–4.60	< 0.001	2.88	1.76–4.69	< 0.001	2.74	1.66–4.51	< 0.001
Health literacy	1.85	1.22–2.83	0.004	1.57	1.01–2.44	0.043	1.57	1.01–2.45	0.04	1.6	1.02–2.51	0.04
Knowledge about screening test	3.76	1.65–8.58	0.002	3.12	1.33–7.30	0.009	3.38	1.46–7.81	0.004	3.37	1.45–7.82	0.005
Perceived susceptibility				1.03	1.91–1.17	0.55						
Perceived severity				0.91	0.80–1.05	0.23						
Perceived benefits				1.21	1.07–1.36	0.002						
Perceived barriers				0.95	0.92–0.98	0.006						
Perceived self-efficacy				0.98	0.91–1.05	0.596						
Attitude							0.88	0.81–0.96	0.007			
Perceived subjective norm							0.97	0.88–1.07	0.59			
Perceived behavioural control							1	0.96–1.04	0.94			
Affect										0.84	0.75–0.94	0.003
Utility										1.2	1.07–1.35	0.002
Subjective norm										0.97	0.89–1.06	0.58
Habit										0.78	0.53–1.15	0.21

*Model 1: R*^*2*^ = *0.158,R*^*2*^*(adj)* = *0.150, Hosmer–Lemeshow goodness-of-fit test: χ*^*2*^ = *1.694, df* = *5, p* = *0.89*

*Model 2: R*^*2*^ = *0.192,R*^*2*^*(adj)* = *0.178, Hosmer–Lemeshow goodness-of-fit test: χ*^*2*^ = *4.61, df* = *8, p* = *0.79*

*Model 3: R*^*2*^ = *0.182, R*^*2*^* (adj)* = *0.171, Hosmer–Lemeshow goodness-of-fit test: χ*^*2*^ = *8.6 df* = *8, p* = *0.37*

*Model 4: R*^*2*^ = *0.201, R*^*2*^* (adj)* = *0.188, Hosmer–Lemeshow goodness-of-fit test: χ*^*2*^ = *15.49, df* = *8, p* = *0.05*

A logistic regression model with the TPB constructs of attitude, subjective norm and perceived behavioural control added to the significant socio-demographic variables also has a good fit (Hosmer–Lemeshow goodness-of-fit test: χ^2^ = 8.6 df = 8, *p* = 0.37) and explains 18.2% of the variance in screening intention (adjusted R^2^ = 0.171), compared to the socio-demographic predictors alone. The addition of the TPB components thus increases the variance by 2.1% when adjusted for other factors. Of the model components, attitude is the only significant predictor among the TPB constructs (β = − 0.119, *p* < 0.05), with positive attitude being associated with positive intention to screen. Subjective norms and perceived behavioural do not significantly predict screening intention. Again, the addition of past screening behaviour shows no significant increase in the variance explained by the TPB (R^2^ = 0.183, adjusted R^2^ = 0.170).

The fourth model, in which the constructs of the TCSB (affect, utility, subjective norms and habit) were added to the regression along with the significant socio-demographic variables, explained 20.1% of the variance in screening intention (adjusted R^2^ = 0.19), but only marginally reached a significant fit (*p* = 0.05). The TCSB components alone explained 3.8% of the variance when adjusted for other factors. Of the model, the affect (β = − 0.173, *p* < 0.005) and utility (β = 0.186, *p* < 0.005) dimensions are the only ones that significantly contribute to the prediction of screening intention, with lower affect and higher utility being associated with positive screening intention.

## Discussion

Cervical cancer screening uptake in this context is influenced by several factors. It could be health system-related [39] or beneficiary related including socio-demographic characteristics, the organisation and accessibility of the health system, and psychological (cognitive, emotional and relational) characteristics [18]. While sociodemographic factors like socioeconomic status, age, education, or employment cannot easily be modified, psychological factors like knowledge about disease and screening or cognitive barriers like beliefs and perceptions are more easily modifiable. Several studies have used health behaviour theories to explore the influence of these socio-cognitive factors on CCS uptake, but very few have compared and/or combined different models in trying to assess their capacity to predict CCS uptake. This study explored the determinants of CCS intention using three different health behaviour theories in combination with a series of socio-economic barriers to screening and knowledge, allowing to compare their capacity to explain screening intention.

Among the socio-demographic variables, accessibility to screening centres was the most significant predictor of screening intention along with the ability of women to make their own decisions with regard to health care expenditure and having a health insurance. These barriers are similar to those found in other studies performed in Taiwan [40] and Kenya [41], where the role of health insurance and male-decision making in cervical cancer screening uptake were observed. The World Health Survey has also confirmed that inaccessibility to health care is a significant barrier to pelvic examination especially for women in LMICs [42].

Another important barrier to CCS participation intention is limited knowledge. According to our findings, the average level of knowledge about cervical cancer and CCS among the women in the participant group was low, confirming findings of related studies conducted in India [43]. Poor knowledge about disease and screening has also been highlighted by others as a primary factor influencing women in LMICs [42] including those with a higher risk for the disease [44]. It is important to know that knowledge about screening plays an important role in decision making and may affect all the other psycho-social factors, that impact on screening, as it serves as a basis for beliefs and perceptions. Using culturally relevant information to educate women about cervical cancer could thus be useful to improve cervical cancer screening uptake, as has been demonstrated elsewhere [45].

Evidence also shows that there is an association between health literacy and knowledge about CCS [46]. Although only very few studies have explored the health literacy of Indians, those that have, have shown poor health literacy levels among the population [47, 48]. This study used a validated tool that measured the participants’ health literacy in the sense of their ability to find, understand, process and apply health-related information, and found a large share of the participants had inadequate health literacy. This could affect their information-seeking behaviour [49]. Whereas there are very few studies that have explored the role of health literacy in CCS behaviour [50], our research shows that women with adequate health literacy have better knowledge about cervical cancer than those with lower levels. This finding has important implications for practice: while the Government of India has launched a health portal with information on diseases like cervical cancer and its prevention with the aim to improve the health knowledge and behaviours of citizens [51], this might not be very effective if the target groups do not know how to access, understand, appraise and apply this information. Also, a majority of individuals especially in the low socio-economic group are incapable of using the internet to find health-related information. Hence, efforts to increase knowledge and awareness on cervical cancer might need to be done in alternative ways, preferably in communities, and should be done in conjunction with a general improvement of health literacy among younger individuals through schools. Evidence from research in other resource-poor settings has shown that health information can be given more effectively when personalized information through direct contact [52]. This approach would also concur with the World Health Organization’s recognition of the need to engage the educational sector to promote health literacy early in life [53] to enhance peoples’ capacities to make healthy decisions and prevent several illness.

Screening for cervical cancer in India is done in an opportunistic way, often involving private health actors, which involves costs [54]. Along with low awareness about the availability of free screening at public hospitals, as found in this study, this explains why in our study having a health insurance and having the ability to make decisions related to health care expenditure were found to impact screening participation. Roughly one out of four women in our study (27%) reported cost-related barriers to screening participation. On the other hand, women who were able to make decisions about health care expenses themselves had a positive intention for CCS screening uptake. It is worth noting that a large majority of women in our sample (80%) relied on partners or family to make healthcare-related decisions, which emphasizes the need for involvement for an involvement of the partner and of other family members in health promotion and knowledge-based interventions to improve screening [41, 55].

### Psychosocial determinants of cervical screening

Apart from socio-economic barriers, knowledge, and health literacy, participation in cervical screening is also influenced by psychosocial variables [56]. These variables can be better understood and accounted for by utilizing theoretical models. Our study was one of the first to compare different models with respect to their capacity to predict the intention to undergo CCS, showing that all three of the models (HBM, TPB and THCSB) contribute to the prediction of screening intention over and above the perceived barriers and knowledge, but that the Theory of Care Seeking Behaviour adds the highest proportion of variance in screening intention (4.5%) followed by the HBM (2.8%), and the TPB (2.1%), although only HBM and TPB models were significant. As such, these findings confirm but also add to the already available literature on social cognitive models applied to cancer screening [57].

Specifically, it confirms the results of earlier studies showing that the TPB is a good predictor of intentions [34], although the explained variance in CCS intentions found in our study was low compared to the 51 and 11% explained in studies by Bish et al. [34] and by Rogers [29] respectively. This difference is probably due to the fact that the latter studies were both conducted in high income countries with a highly developed healthcare system, which would reduce the relative importance of socio-economic barriers and knowledge as main determinants of screening participation. As in other studies [58, 59], attitude was the best single predictor of CCS intentions from amongst the variables of the TPB, while unlike some other studies [28, 60], subjective norm and PBC were not found to influence intentions in our study. The latter may be due to perceived responses or awareness provided before assessment, or to the way the constructs were measured. Furthermore, past behaviour has been suggested as a good predictor of future behaviour, we added past *CCS behaviour* to the model with TPB but did not find it significantly increased the proportion of explained variance of screening intention. This could be because individuals were unaware of the need for regular cervical screening, or because past screening behaviour could have been related to the presence of symptoms, as has been found in earlier studies. As such, it is better to include past *regular general screening behaviour* as an additional variable with the TPB than past CCS uptake.

Like the TPB, the HBM was also found to significantly explain CCS intention, confirming several other studies [61, 62], although the model has been criticized for its inconsistent use and minimum predictive capacity [63]. From among the HBM constructs, perceived benefits and barriers were the most significant ones, which concurs with the studies conducted earlier [61, 62]. Thus, women who perceive screening to be beneficial and who perceived fewer barriers to participate in screening are more likely to have positive screening intentions. It is also worth noting that the perceived barriers to screening may be health system-related, like the accessibility and cost factors discussed earlier, but may also refer to perceived barriers in the procedure, outcome or time investment.

Finally, our findings showed that of the three socio-cognitive models tested, the TCSB adds the highest proportion of explained variance in CCS intention over and above the perceived barriers and knowledge, but that the model as such does not have a good fit to predict CCS intention. This is at odds with findings from other studies based on the theory, where the constructs of habit, norm and utility were found to be associated with intention to participate in breast cancer screening [64]. In our study, only the dimensions of utility and affect contributed significantly to the intention to be screened, suggesting that screening intention is facilitated by perceived health benefits, yet that emotions like fear, embarrassment or anxiety can be important barriers to screening. This is in accordance with Ajzen’s acknowledgement that emotions and affect are most commonly stated as missing in the TPB [65] and with research showing that anticipated affect reactions can predict behavioural intentions, independent of attitudes regarding the behaviour [66]. Indeed, the inclusion of the affect variable of the TCSB into the model with TPB constructs increases the additional explained variance slightly from 2.1 to 2.3% as seen in this study. Thus, it is worth noting that, like past general screening behaviour, an individual’s emotions like fear and anxiety can make a significant contribution and improve the often-used models like the TPB.

These psychosocial barriers experienced by women identified were at individual level. And majority of the individual barriers were related to negative attitude towards screening (37.7%) followed by perceptions and superstitious beliefs about disease (28.8%). Nearly 50% of the women were anxious, shy or afraid of procedure. Besides, interpersonal factors like lack of support from husband or family, and socio-cultural factors like stigma related to cancer also affected acted as barriers. Furthermore, structural barriers, like poor accessibility, lack of time or cost, long waiting hours or screening process were faced by women.

### Implications for practice

Health behaviour theories add to our understanding of the psychosocial factors that underlie intentions and behaviour related to health. As such, they can inform interventions that aim to influence health behaviours. However, as several models have been developed, validated and used, the question arises as to which model performs best at explaining health behaviour. The current study revealed that while some of the models are slightly better at explaining cervical cancer uptake or the intention to participate in cancer screening, it also showed that certain constructs of the three models contribute more to explaining this behaviour than others. As such, it seems indicated to combine constructs from different theories for designing interventions. More specifically, to improve women’s intention to screen it is important to enhance their positive attitude towards screening, highlight the perceived benefits and reduce perceived barriers. As such, this research provides a scope for integrating different theoretical models with a view to better explain screening behaviour and improve the effectiveness of interventions.

### Limitations

This study is not without limitations. It is possible that the results were influenced by social desirability bias, when participants were asked for their behaviour or intention. It is also possible that bias was induced during data collection due to the way participants perceived the questions. Lastly, it should be recognized that this study considered screening intentions, and not actual screening behaviour, as a main outcome variable, since only a very small proportion of the sample regularly participated in screening. Intentions may not always result in behaviour, although it can be considered as an important determinant and proxy. However, despite these limitations, the comparison of three distinct social-cognitive theories to predict CCS intention, in combination with questions regarding socio-economic barriers, knowledge, and health literacy, draws attention to the psychosocial factors that predict CCS uptake. Since these factors are modifiable they can be addressed to improve cervical cancer screening uptake, whilst taking account of the structural and health systems related barriers and the role of health literacy on the cognitive determinants of screening uptake.

## Conclusion

Although the applicability of theoretical models may vary across contexts, this study is one of the first to compare three different health behaviour theories with regard to their capacity to predict the intention of women to participate in cervical cancer screening. We found that in addition to socio-economic barriers, knowledge, and health literacy, both the HBM and the TPB can be used to predict women’s screening intentions, while the TCSB fails to predict screening intentions as a model, yet several of its dimensions contribute significantly to screening intentions. The latter suggests that it in order to optimise the prediction of screening participation it may be necessary to the constructs from different theoretical models. Specifically, it was shown that the constructs of attitude towards screening as defined by the TPB, perceived benefits about screening and barriers deriving from the HBM and affect from TCSB are the best predictors of screening intention. In addition, it was noted that many structural or health system related barriers towards screening are not considered in socio-cognitive models. As such barriers play an important role in screening uptake, especially in resource-poor settings, they should be added to enhance the understanding of what moves women to participate in screening and addressed in efforts to improve screening rates. In that regard, there remains a need to promote knowledge about the disease and about the benefits of screening and its availability, in addition to reducing negative attitudes towards screening and addressing negative emotions.

## Data Availability

The datasets generated and/or analysed during the current study are not publicly available due to ethical reasons but are available from the corresponding author on reasonable request.
